# Differences in Characteristics and Outcomes Between Large-Cell Neuroendocrine Carcinoma of the Ovary and High-Grade Serous Ovarian Cancer: A Retrospective Observational Cohort Study

**DOI:** 10.3389/fonc.2022.891699

**Published:** 2022-05-04

**Authors:** Li Pang, Zhiqiang Guo

**Affiliations:** Department of Obstetrics and Gynecology, Shengjing Hospital of China Medical University, Shenyang, China

**Keywords:** ovarian large-cell neuroendocrine carcinoma, high-grade serous ovarian cancer, overall survival, cancer-specific survival, SEER database

## Abstract

**Background:**

Owing to its extremely low incidence and the paucity of relevant reports, there is currently no recognized first-line treatment strategy for ovarian large-cell neuroendocrine carcinoma, and there are no statistics related to prognosis derived from large samples. This study aimed to investigate the characteristics, outcomes, and independent predictors of survival for ovarian large-cell neuroendocrine carcinoma and compare them with those of high-grade serous ovarian cancer.

**Methods:**

The Surveillance, Epidemiology, and End Results database was used to identify women diagnosed with ovarian large-cell neuroendocrine carcinoma or high-grade serous ovarian cancer from 1988 to 2015. Clinical, demographic, and treatment characteristics were compared between the groups. Propensity-score matching, Cox risk regression analysis, and Kaplan–Meier survival curves were used to analyze the data.

**Results:**

In total, 23,917 women, including 23,698 (99.1%) diagnosed with high-grade serous ovarian cancer and 219 (0.9%) diagnosed with ovarian large-cell neuroendocrine carcinoma, were identified. Age >77 years, diagnosis before 2003–2010, and advanced-stage disease were more common in patients with ovarian large-cell neuroendocrine carcinoma than in those with high-grade serous ovarian cancer. Women with ovarian large-cell neuroendocrine carcinoma were less likely to receive adjuvant chemotherapy (54.8% vs. 81.9%) but more likely to receive radiotherapy (3.2% vs. 1.5%; both P<0.001) than women with high-grade serous ovarian cancer. Stage, chemotherapy, and tumor size were independent predictors of overall survival, and the risk of death was greater in the advanced stage than in the early stage (P=0.047). Chemotherapy and tumor size were also independent predictors of cancer-specific survival. Overall and cancer-specific survival rates were significantly low for ovarian large-cell neuroendocrine carcinoma than for more malignant high-grade serous ovarian cancer.

**Conclusions:**

Compared to patients with high-grade serous ovarian cancer, those with ovarian large-cell neuroendocrine carcinoma presented more often with advanced-stage disease and had decreased overall and cancer-specific survival rates.

## Introduction

Neuroendocrine tumors, an aggressive form of cancer, develop from neuroendocrine cells and often occur in the gastrointestinal tract, pancreas, and lungs. They are rarely found in other tissues and organs, and reports of them in the female reproductive tract are rare (1). Patients with neuroendocrine tumors often exhibit symptoms of Cushing’s syndrome (2).

Currently, studies regarding the origin of ovarian neuroendocrine tumors are few. Nonetheless, ovarian neuroendocrine tumors can be roughly classified into carcinoid tumors, atypical carcinoid tumors, small-cell carcinoma of the ovary (SCCO), and large-cell neuroendocrine carcinoma of the ovary (LCNEO) (3). According to World Health Organization (WHO) guidelines, non-small-cell neuroendocrine carcinoma (NSCNEC) is synonymous with LCNEC (3). Carcinoid and atypical carcinoid tumors are classified as low-grade neuroendocrine tumors, whereas SCCO, LCNE8O, and NSCNEC are classified as high-grade neuroendocrine carcinomas. Primary LCNEO is a highly malignant and aggressive disease with poor prognosis. In addition, metastasis and recurrence can easily occur even in the early stages of the disease. Primary LCNEO is often accompanied by the presence of other epithelial and germ cell tumors (4–6), and the incidence of simple LCNEO is sporadic (7–11). As of December 2019, only 18 records of pure LCNEO were available on PubMed (12).

Although most patients receive surgical treatment and postoperative adjuvant chemotherapy, the prognosis of LCNEO remains extremely poor (12). The poor prognosis of patients despite early diagnosis demonstrates the high biological aggressiveness of this tumor. Owing to the extremely low incidence of LCNEO and the paucity of relevant reports in the literature, there is currently no recognized first-line treatment strategy, and there are no statistics related to prognosis in large samples.

We aimed to investigate the natural course, treatment options, and outcomes of LCNEO. The overall survival (OS) and cancer-specific survival (CSS) rates were calculated using data from a population-based tumor registry. These data were also used to identify independent predictors of LCNEO. Furthermore, although serous ovarian cancer is a common subtype of ovarian cancer with a high potential for malignancy and poor prognosis, we compared LCNEO with high-grade serous ovarian cancer (HG-GOC) because SCCO, LCNEO, and NSCNEC are classified as high-grade neuroendocrine carcinomas. To date, no population-based study has compared the outcomes of LCNEO and HG-SOC. Therefore, we compared clinical characteristics and outcomes between patients with LCNEO and those with HG-SOC to provide a reference for clinical treatment.

## Materials and Methods

### Eligibility Criteria and Data Collection

**Figure 1:**
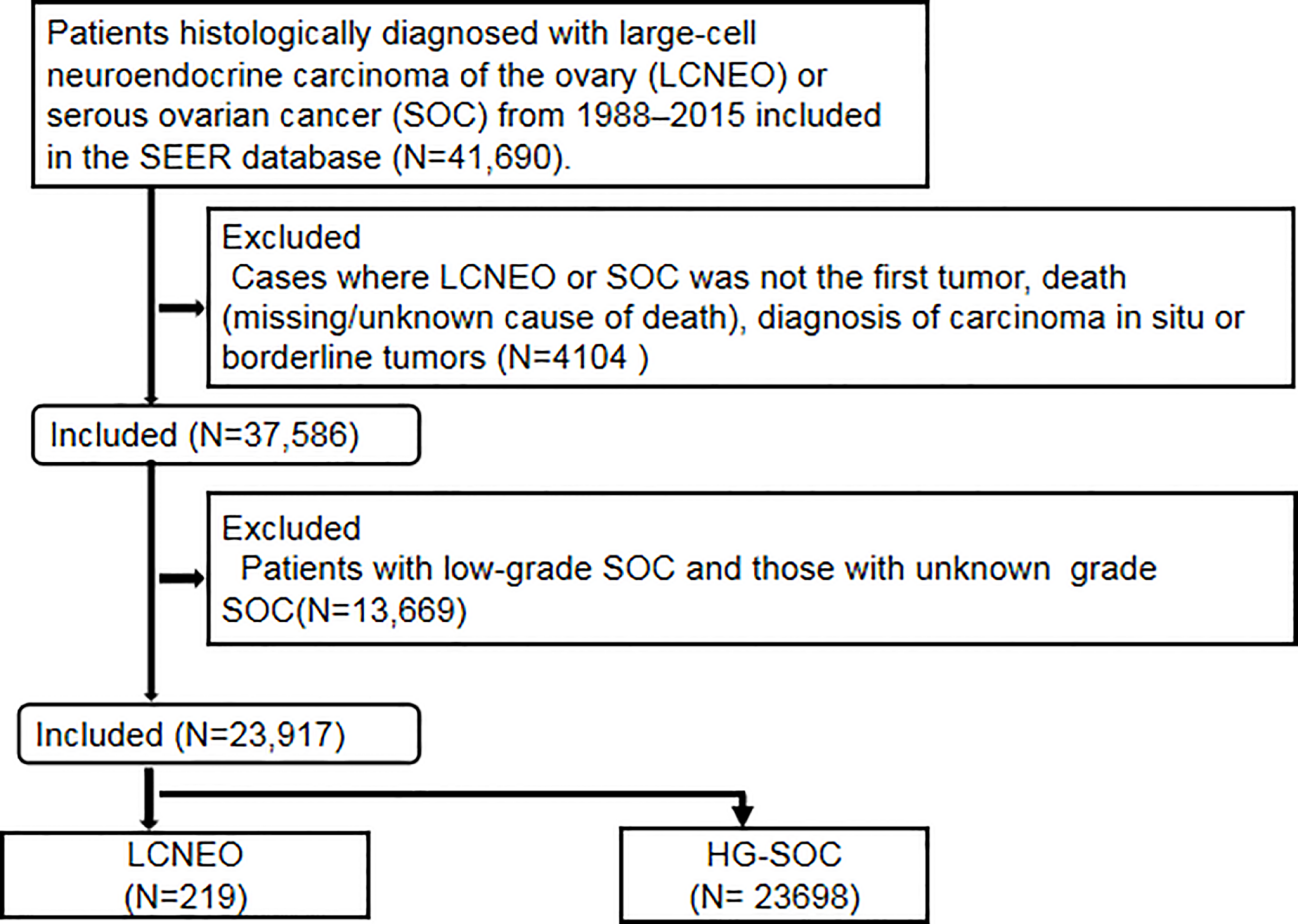
Study flow chart.

This retrospective observational cohort study utilized the Surveillance, Epidemiology, and End Results (SEER) database of the National Cancer Institute (13). The SEER program is the largest population-based tumor registration system in the United States (US). Launched in 1973, the SEER database has operated for more than 40 years and currently covers approximately 34.6% of the US population. It is considered a powerful tool for identifying population characteristics and studying the long-term prognosis of rare tumors. Patients histologically diagnosed with LCNEO or HG-SOC between 1988 and 2015 were identified in the SEER database as follows: Site code: primary malignant tumor in the ovary (ICD-O-3/WHO 2008). The inclusion criteria were as follows: histology code: large-cell carcinoma (8012/3), large-cell neuroendocrine carcinoma (8013/3), non-small-cell carcinoma (8046/3), serous cystadenocarcinoma (8441/3), and papillary serous cystadenocarcinoma (8460/3). The exclusion criteria were as follows: diagnosis of carcinoma *in situ* or borderline tumors, cases where LCNEO or SOC was not the first tumor, death (missing/unknown cause of death), and low-grade and unknown-grade SOCs (**Figure 1**). Notably, the SEER database has certain limitations. For example, it provides information on whether the patient received chemotherapy but not on the number of chemotherapy cycles, chemotherapy regimens, or neoadjuvant chemotherapy regimens received. It also does not specify the time and location of recurrence or have patients’ preoperative imaging data.

The diagnostic grades recorded in the SEER database are in accordance with the guidelines of the American Joint Committee on Cancer (AJCC) and the International Federation of Gynecology and Obstetrics and are as follows: G1, well-differentiated; G2, moderately differentiated; G3, poorly differentiated; and G4, undifferentiated. The AJCC pairs G3 and G4. In this study, the SOC grades were classified into two-tiers—low grade (G1, G2) and high grade (G3, G4)—following expert recommendations for diagnostic practice (14, 15).

The de-identified data in the SEER database are publicly available; thus, their use was exempt from review by the Institutional Review Board of Shengjing Hospital Affiliated with China Medical University. The requirement for informed consent was also waived.

**Figure 2:**
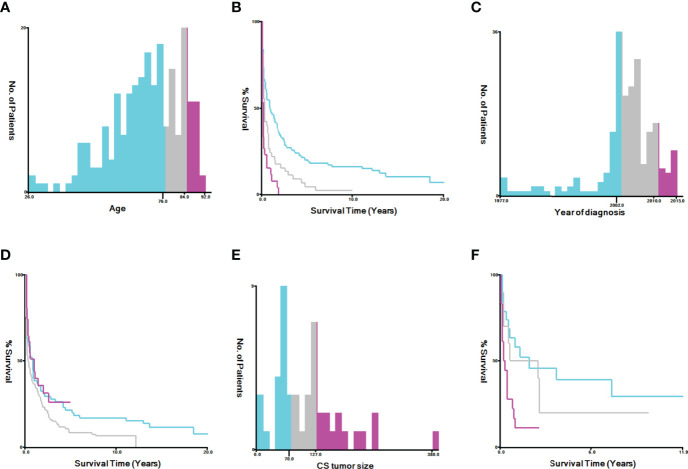
Identification of optimal cut-off values for age [**(A)** best cut-off value for age, **(B)** survival curves for different ages, **(C)** best cut-off value for tumor size, and **(D)** survival curves for different tumor sizes] and year of diagnosis [**(E)** best cut-off value for tumor size, **(F)** survival curves for different tumor sizes] *via* X-tile software analysis. The optimal minimum and maximum cut-off values for age (based on overall survival) are 76 and 84 years, those for tumor size are 70 and 127 mm, and those for the year of diagnosis are 2002 and 2010, respectively.

SEER*Stat 8.3.8 software (https://seer.cancer.gov/data/) was used to generate case listings and record patient information, including demographics, clinical pathology, and treatment parameters. Staging information was determined based on the AJCC staging system. X-tile software (Yale University, New Haven, CT, USA) was used to assess the optimal cut-off values for age, tumor size, and year of diagnosis (**Figure 2**). The best minimum and maximum cut-off values for age were 76 and 85 years, respectively, while the best minimum and maximum cut-off values for tumor size were 70 mm and 127 mm, respectively. The best cut-off values for the year of diagnosis were 2002 and 2010.

### Clinical and Demographic Characteristics

We analyzed patient demographic data, including age at diagnosis (grouped into ≤76, 77–84, and ≥85 years), race (white, black, other, and unknown), marital status (single/unmarried, married, divorced/separated, widowed, and unknown), insurance status (insured, any form of Medicaid, uninsured, and unknown), AJCC stage (I+II, III+IV, and unknown), and year of diagnosis (≤2002, 2003–2010, and 2011–2015). Further, we assessed clinical characteristics, including grade (poorly differentiated, undifferentiated, and unknown), lymph node status (negative, positive, not examined, and unknown), tumor size (≤70, 70–127, and ≥128 mm), cancer antigen 125 status (negative, positive, and unknown), and laterality (left, right, unilateral with side unspecified, bilateral [single primary], and paired site with no laterality information). In addition, we assessed treatment patterns, including surgery (yes, no, and unknown), chemotherapy (yes and no), and radiotherapy (yes and no). Data on time of follow-up after diagnosis, life status, and cause of death were collected from the database to evaluate survival after the disease (i.e., CSS) and OS.

### Statistical Analysis

Chi-squared or Fisher’s exact test was used to compare clinical and demographic characteristics between patients with LCNEO and those with HG-SOC. Categorical data are presented as numbers and percentages (N, %). Optimal cut-off values for age, tumor size, and year of diagnosis were assessed using X-tile software (**Figure 2**). Propensity-score matching and multivariate analysis were used to evaluate OS and CSS for the LCNEO and HG-SOC groups. Univariate and multivariate Cox risk regression analyses were used to determine independent predictors of OS and CSS in patients with LCNEO. OS and CSS rates were calculated using Kaplan–Meier curves, and the log-rank test was used for comparison. All data were analyzed using IBM SPSS Statistics for Windows/Macintosh, ver. 23 (IBM Corp., Armonk, NY, USA). GraphPad Prism 8.3.0 (GraphPad Software, San Diego, CA, USA) was used to plot Kaplan–Meier survival curves, and P-values <0.05 were considered statistically significant.

## Results

We identified 219 cases of LCNEO from the SEER database of the National Cancer Institute that met our eligibility criteria (13). Compared to patients with HG-SOC, those with LCNEO were more likely to be aged 77–84 years (20.5% vs. 11.2%) and ≥85 years (13.7% vs. 2.9%; P<0.0001). They were also more likely to be black (10.5% vs. 6.0%; P=0.043), divorced (35.2% vs. 16.3%; P<0.0001), and have stage III–IV disease (P=0.044). LCNEO was also more likely to be diagnosed between 2003 and 2010 than HG-SOC (64.4% vs. 36.9%; P<0.0001). Lack of lymphadenectomy was more common in patients with LCNEO than in those with HG-SOC (73.5% vs. 47.4%; P<0.001). Patients with LCNEO received radiotherapy more often than those with HG-SOC (3.2% vs. 1.5%; P=0.035; **Table 1**).

**Table 1:** Demographic and clinical characteristics comparing LCNEO and HG-SOC (pre-matching and pos-matching).

Subject characteristics	pre-matching		*P-value*	pos-matching		*SD*
	LCNCO	HG-SOC		LCNCO	HG-SOC	
	N (%)	N (%)		N (%)	N (%)	
ALL	219 (0.9)	23698 (99.1)		197	197	
Age at diagnosis						
Mean age (years,SD)	69.79 (±13.54)	62.09 (±11.58)	*P-value*	68.72 (±12.44)	61.11 (±10.96)	
**Age at diagnosis (years)**						
≤76	144 (65.8)	20349 (85.9)	<0.0001	118 (59.9)	119 (60.4)	0.03
77-84	45 (20.5)	2664 (11.2)		46 (23.4)	48 (24.4)	
≥85	30 (13.7)	685 (2.9)		33 (16.7)	30 (15.2)	
**Race**			0.043			
White	181 (82.6)	20542 (86.7)		170 (86.3)	174 (88.3)	0.002
Black	23 (10.5)	1418 (6.0)		18 (9.1)	19 (9.6)	
Other	15 (6.9)	1700 (7.2)		9 (4.6)	4 (2.1)	
Unknown	0 (0)	38 (0.1)		0 (0.0)	0 (0.0)	
**Marital status**			<0.0001			0.045
Single/unmarried	22 (10.0)	3162 (13.3)		21 (10.7)	23 (11.7)	
Married	92 (42.0)	13253 (55.9)		89 (45.2)	88 (44.6)	
Divorced/separated	23 (10.5)	2719 (11.5)		13 (6.6)	10 (5.1)	
Widowed	77 (35.2)	3851 (16.3)		71 (36.0)	71 (36.1)	
Unknown	5 (2.3)	713 (3.0)		3 (1.5)	5 (2.5)	
**Insurance status**			<0.0001			0.028
Insured	51 (23.3)	10004 (42.2)		49 (24.9)	48 (24.4)	
Any Medicaid	7 (3.2)	1148 (4.9)		4 (2.0)	2 (1.0)	
Uninsured	8 (3.7)	361 (1.5)		3 (1.5)	6 (3.0)	
Unknown	153 (69.9)	12185 (51.4)		141 (71.6)	141 (71.6)	
**Stage**			***0.044***			0.042
I+II	10 (4.6)	2164 (9.13)		7 (3.6)	12 (6.1)	
III+IV	113 (51.6)	11218 (47.4)		105 (53.3)	103 (52.3)	
Unknown	96 (43.8)	9316 (39.3)		85 (43.1)	82 (41.6)	
**Year of diagnosis**			<0.0001			0.036
≤2002	47 (21.5)	8024 (33.9)		37 (18.8)	38 (19.3)	
2003-2010	141 (64.4)	8750 (36.9)		136 (69.0)	129 (65.5)	
2011-2015	31 (14.1)	6924 (29.2)		24 (12.2)	30 (15.2)	
**Grade**			<0.0001			
Poorly differentiated	62 (28.3)	16452 (69.4)		54 (27.4)	52 (26.4)	0.097
Undifferentiated	43 (19.6)	7246 (30.6)		37 (18.8)	145 (73.6)	
Unknown	114 (52.1)	0 (0.0)		106 (53.8)	0 (0)	
**Lymph nodes status**			<0.0001			0.012
Negative	17 (7.7)	5168 (21.8)		17 (8.6)	16 (8.1)	
Positive	21 (9.6)	5619 (23.7)		17 (8.6)	19 (9.6)	
No examined	161 (73.5)	11304 (47.7)		151 (76.6)	149 (75.6)	
Unknown	20 (9.2)	1607 (6.8)		12 (6.2)	13 (6.7)	
**Laterality**			<0.0001			0.045
Left	48 (21.9)	4403 (18.6)		43 (21.8)	45 (22.8)	
Right	41 (18.7)	4732 (20.0)		35 (17.8)	38 (19.3)	
Only one side - side unspecified	5 (2.3)	124 (0.5)		3 (1.5)	1 (0.5)	
Bilateral, single primary	45 (20.6)	13423 (56.6)		43 (21.8)	45 (22.8)	
Paired site, but no information concerning laterality	80 (36.5)	1016 (4.3)		73 (37.1)	68 (34.6)	
**Tumor size**			<0.0001			0.021
≤70	19 (8.7)	4377 (18.5)		18 (9.1)	14 (7.1)	
71-127	10 (4.6)	4095 (17.3)		9 (4.6)	10 (5.0)	
≥128cm	18 (8.2)	6229 (26.2)		11 (5.6)	12 (6.1)	
Unknown	172 (78.5)	8997 (38.0)		159 (80.7)	161 (81.7)	
**Ca125**			0.057			0.015
Negative	89 (40.6)	11478 (48.4)		73 (37.1)	75 (38.1)	
Positive	13 (6.0)	1438 (6.1)		9 (4.5)	4 (2.0)	
Unknown	117 (53.4)	10782 (45.5)		115 (58.4)	118 (59.9)	
**Surgery performed**			<0.0001			0.013
Surgery	141 (64.3)	22729 (95.9)		135 (68.5)	133 (67.5)	
No surgery	77 (35.2)	918 (3.9)		61 (31.0)	60 (20.5)	
Unknown	1 (0.5)	51 (0.2)		1 (0.5)	4 (2.0)	
**Chemotherapy**						0.011
Yes	120 (54.8)	19413 (81.9)	<0.0001	116 (58.9)	117 (59.4)	
No	99 (45.2)	4285 (18.1)		81 (41.1)	80 (40.6)	
**Radiotherapy**			***0.035***			
Yes	7 (3.2)	347 (1.5)		5 (2.5)	2 (1.0)	0.05
No	212 (96.8)	23351 (98.5)		192 (97.5)	195 (99.0)	

LCNEO, large-cell neuroendocrine carcinoma of the ovary; HG-SOC, high-grade serous ovarian cancer.

Bold means p < 0.05.

In univariate regression models that were restricted to women with LCNEO, age at diagnosis (in years), stage, lymph node status, tumor size, CA125, laterality, surgery, and chemotherapy were independent prognostic factors for OS and CSS. In multivariate Cox regression analysis, stage, chemotherapy, and tumor size were identified as independent predictors of OS (**Tables 2** and **3**), while chemotherapy and tumor size were identified as independent predictors of CSS (**Tables 2** and **3**).

**Table 2:** Univariable Cox Regression for analyzing the associated factors for developing large cell carcinoma of the ovarian (LCNEO).

Subject characteristics	Overall survival (OS)		Cancer-specific survival (CSS)	
	HR (95%CI)	*P*-value	HR (95%CI)	*P*-value
**Age at diagnosis (years)**				
≤76	Reference		Reference	
77-84	1.715 (1.208-2.436)	**0.003**	1.751 (1.222-2.510)	**0.002**
≥85	3.008 (1.964-4.608)	**<0.002**	3.114 (2.015-4.814)	**<0.001**
**Race**				
White	Reference			
Black	1.099 (0.697-1.734)	0.683	1.206 (0.765-1.910)	0.417
Other	0.735 (0.408-1.323)	0.304	0.681 (0.359-1.292)	0.240
Unknown	NA	NA	NA	NA
**Marital status**				
Single/unmarried	Reference		Reference	
Married	0.947 (0.566-1.583)	0.835	1.067 (0.612-1.863)	0.818
Divorced/separated	1.470 (0.781-2.766)	0.232	1.626 (0.831-3.180)	0.156
Widowed	1.604 (0.954-2.697)	0.075	1.744 (0.993-3.061)	0.053
Unknown	NA	NA	NA	NA
**Insurance status**				
Insured				
Any Medicaid	1.884 (0.840-4.226)	0.125	1.357 (0.533-3.455)	0.522
Uninsured	1.592 (0.711-3.563)	0.258	1.589 (0.710-3.560)	0.260
Unknown	1.014 (0.71-1.446)	0.938	0.953 (0.667-1.362)	0.792
**Stage**				
I+II	Reference		Reference	
III+IV	2.483 (1.083-5.695)	**0.032**	3.476 (1.273-9.491)	**0.015**
Unknown	NA	NA	NA	NA
**Year of diagnosis**				
≤2002	Reference	0.077	Reference	0.102
2003-2010	1.384 (0.971-1.974)	0.072	1.371 (0.947-1.984)	0.094
2011-2015	0.933 (0.548-1.587)	0.797	0.929 (0.538-1.604)	0.791
**Grade**				
Poorly differentiated	Reference		Reference	
Undifferentiated	0.887 (0.557-1.414)	0.615	0.920 (0.568-1.490)	0.735
Unknown	NA	NA	NA	NA
**Lymph nodes status**				
Negative	Reference		Reference	
Positive	3.474 (1.418-8.512)	**0.006**	5.259 (1.738-15.911)	**0.003**
No examined	6.347 (2.935-13.727)	**<0.001**	9.607 (3.523-26.198)	**<0.001**
Unknown	NA	NA	NA	NA
**Surgery performed**				
Surgery	Reference		Reference	
No surgery	3.565 (2.588-4.910)	**<0.001**	3.555 (2.554-4.948)	**<0.001**
Unknown	NA	NA	NA	NA
**Chemotherapy**				
Yes	Reference		Reference	
No	3.575 (2.625-4.869)	**<0.001**	3.419 (2.490-4.694)	**<0.001**
**Radiotherapy**				
Yes	Reference		Reference	
No	2.197 (0.815-5.921)	0.120	2.082 (0.772-5.615)	0.148
**Tumor size**				
≤70	Reference		Reference	
71-127	1.348 (0.549-3.307)	0.514	1.466 (0.588-3.656)	0.412
≥128cm	3.056 (1.385-6.744)	**0.006**	2.785 (1.219-6.363)	**0.015**
Unknown	NA	NA	NA	NA
**Ca125**				
Negative	Reference		Reference	
Positive	0.433 (0.207-0.905)	**0.026**	0.406 (0.185-0.890)	**0.024**
**Laterality**				
Left	Reference		Reference	
Right	1.359 (0.828-2.231)	0.224	1.335 (0.794-2.247)	0.276
Only one side - side unspecified	1.800 (0.634-5.110)	0.270	1.899 (0.666-5.415)	0.230
Bilateral, single primary	1.720 (1.058-2.780)	**0.029**	1.715 (1.036-2.839)	**0.036**
Paired site, but no information concerning laterality	2.769 (1.824-4.201)	**<0.001**	2.623 (1.704-4.038)	**<0.001**

HR, Hazard Ratio; CI, Confidence Interval; NA, Not available.

Bold means p < 0.05.

**Table 3:** Multivariate Cox Regression for analyzing the associated factors for developing large cell carcinoma of the ovarian (LCNEO).

Subject characteristics	Overall survival (OS)		Cancer-specific survival (CSS)	
	HR (95%CI)	*P*-value	HR (95%CI)	*P*-value
**Age at diagnosis (years)**				
≤76	Reference	0.380	Reference	0.348
77-84	1.274 (0.211-7.691)	0.792	1.291 (0.210-7.961)	0.783
≥85	0.276 (0.041-1.876)	0.188	0.245 (0.033-1.798)	0.167
**Stage**				
I+II	Reference		Reference	
III+IV	9.714 (0.896-17.21)	**0.047**	4.346 (1.246-12.15)	0.974
Unknown	NA	NA	NA	NA
**Lymph nodes status**				
Negative	Reference	0.312	Reference	0.381
Positive	0.261 (0.015-4.490)	0.355	0.290 (0.016-5.307)	0.404
No examined	0.734 (0.058-9.216)	0.810	0.810 (0.065-10.182)	0.871
Unknown	NA	NA	NA	NA
**Surgery performed**				
Surgery	Reference		Reference	
No surgery	2.048 (0.721-5.820)	0.178	2.066 (0.717-5.954)	0.179
Unknown	NA	NA	NA	NA
**Chemotherapy**				
Yes	Reference			Reference
No	8.693 (2.308-32.750)	**0.001**	12.970 (3.206-52.464)	**<0.001**
**Tumor size**				
≤70	Reference		Reference	
71-127	2.312 (0.479-11.153)	0.297	3.239 (0.606-17.318)	0.169
≥128cm	4.714 (1.423-15.618)	**0.011**	5.388 (1.496-19.404)	**0.010**
Unknown	NA	NA	NA	NA
**Ca125**				
Negative	Reference		Reference	
Positive	0.986 (0.187-5.208)	0.986	1.112 (0.203-6.103)	0.903
Unknown	NA	NA	NA	NA
**Laterality**				
Left	Reference	0.466	Reference	0.248
Right	1.791 (0.467-6.866)	0.395	2.815 (0.659-12.020)	0.162
Only one side - side unspecified	12.18 (0.564-163.187)	0.111	21.886 (0.878-45.751)	0.060
Bilateral, single primary	0.658 (0.141-3.062)	0.594	0.708 (0.142-3.518)	0.673
Paired site, but no information concerning laterality	1.878 (0.376-9.365)	0.442	2.274 (0.393-13.138)	0.359

HR, Hazard Ratio; CI, Confidence Interval; LCNEO, large cell carcinoma of the ovarian; NA, Not available.

Bold means p < 0.05.

**Figure 3:**
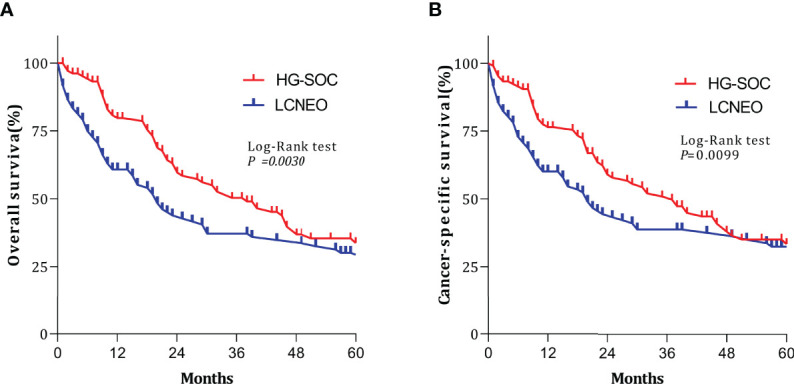
Survival outcomes following propensity-score matching. **(A)** Overall survival and **(B)** cancer-specific survival based on tumor types. Log-rank tests were used to generate P-values. HG-SOC, high-grade serous ovarian cancer; LCNEO, large-cell neuroendocrine carcinoma of the ovary.

After propensity-score matching, we evaluated survival in 394 patients (LCNEO group, n=197; HG-SOC group, n=197). Demographic characteristics were well-balanced between the two groups (all standard deviations ≤0.10; **Table 1**). The median OS for LCNEO was 20 months, while that for HG-SOC was 38 months (hazard ratio [HR], 1.420; 95% confidence interval [CI], 1.022–1.974; P=0.003; **Figure 3A**). The median CSS for LCNEO was 19 months, while that for HG-SOC was 37 months (HR, 1.285; 95% CI, 0.916–1.801; P=0.0099; **Figure 3B**). Women with LCNEO had significantly lower OS and CSS rates than those with HG-SOC (3-year OS rates: 37.1% vs. 50.2%, net difference: 13.1%, P=0.003; **Figure 3A**; 5-year OS rates: 28.7% vs. 35.3%, net difference: 6.6%, P=0.003; **Figure 3A**; 3-year CSS rates: 38.6% vs. 50.8%, net difference: 12.2%, P=0.0099; **Figure 3B**; 5-year CSS rates: 32.3% vs. 34.9%, net difference: 1.6%, P=0.0099; **Figure 3B**). OS and CSS were worse in patients with LCNEO than in those with HG-SOC (P<0.001).

Multivariable analysis revealed that LCNEO was associated with a 1.4-fold increased risk of death from ovarian neoplasm when compared with HG-SOC (adjusted HR, 1.408; 95% CI, 1.004–1.976; P=0.047; **Table 4**). Tumor size, stage, and chemotherapy also remained independent prognostic factors for OS (all adjusted P<0.05). LCNEO remained an independent prognostic factor associated with decreased CSS when compared with HG-SOC. In addition,LCNEO was associated with a 1.5-fold increased risk of death from ovarian neoplasm when compared with HG-SOC (adjusted HR 1.502; 95% CI, 1.013–1.867; P=0.042; **Table 4**). Moreover, tumor size and chemotherapy remained independent prognostic factors for CSS (all adjusted P<0.05).

**Table 4:** Multivariable analysis of survival outcomes after propensity-score matching.

Subject characteristics	Overall survival (OS)		Cancer-specific survival (CSS)	
	HR (95% CI)	*P*-value	HR (95% CI)	*P* value
**Tumor size**				
≤70				
71–127	1.332 (0.662–2.681)	0.421	1.332 (0.662–2.681)	0.526
≥128 cm	1.859 (1.010–3.419)	**0.046**	1.752 (0.990–3.232)	**0.037**
Unknown	2.401 (1.281–4.500)	**0.006**	2.379 (1.262–4.420)	**0.007**
**Stage**				
I+II				
III+IV	3.317 (1.593–6.906)	**0.001**		
Unknown	1.892 (0.851–4.205)	0.118		
**Chemotherapy**				
Yes				
No	2.851 (2.073–3.922)	**<0.001**	2.753 (2.012–3.902)	**<0.001**
**Tumor type**				
HG-SOC				
LCNEO	1.408 (1.004–1.976)	**0.047**	1.502 (1.013–1.867)	**0.042**

Cox proportional hazard regression models for P-values. All the listed covariates were entered in the final models. Significant P-values are emboldened.

HR, hazard ratio; CI, confidence interval; LCNEO, Large-cell neuroendocrine carcinoma of the ovarian; HG-SOC, high-grade serous ovarian cancers.

Bold means p < 0.05.

## Discussion

This study investigated the natural history, prognosis, and independent predictors of OS and CSS in patients with LCNEO using data from a population-based tumor registry. HG-SOC is a common subtype of ovarian cancer with high malignant potential and poor prognosis. Although LCNEO is relatively rare in clinical practice, some studies have reported its aggressiveness and poor prognosis; however, these studies are majorly case reports or small case series. Because SCCO, LCNEO, and NSCNEC are classified as high-grade neuroendocrine carcinomas, we compared LCNEO and HG-SOC with higher malignant potential to further clarify the malignant degree of LCNEO. Additionally, we compared clinical characteristics and outcomes between patients with LCNECO and those with HG-SOC. Our findings highlight the unique natural history and aggressive clinical course of LCNEO. In patients with LCNEO, advanced disease and tumor size >127 mm were associated with decreased OS, while tumor size >127 mm was associated with decreased CSS. Although chemotherapy significantly reduced the risk of death in patients with LCNEO, OS and CSS were significantly worse among patients with LCNEO than among those with HG-SOC, despite HS-SOC’s greater potential for malignancy. Lack of lymphadenectomy was more common in patients with LCNEO than in those with HG-SOC. Women with LCNEO were less likely to receive adjuvant chemotherapy and more likely to receive radiotherapy than those with HG-SOC.

Our results indicated that compared to patients with HG-SOC, those with LCNEO were more commonly aged >77 years, more often black, divorced, and had stage III–IV disease. In addition, LCNEO was diagnosed more commonly between 2003 and 2010 than HG-SOC. Although these findings indicate that older, divorced, and black patients are more susceptible to LCNEO, the exact reasons for these associations remain unclear.

After comparing rates of radiotherapy (3.2% vs. 1.5%; P=0.035) and chemotherapy (54.8% vs. 81.9%, <0.0001) between patients with LCNEO and those with HG-SOC, we concluded that women with LCNEO were less likely to receive adjuvant chemotherapy but more likely to receive radiotherapy compared to those with HG-SOC. We believe this has something to do with the characteristics and treatment of ovarian neuroendocrine cancer. The treatment plan for HG-SOC often involves a combination of surgery and chemotherapy, and rarely involves radiotherapy. However, in the literature, the comprehensive treatment of LCNEO often involves a combination of surgery, chemotherapy, and radiotherapy to improve the prognosis of patients (12, 16, 17). In addition, because patients with LCNEO were often diagnosed when the disease was at an advanced stage or when they were older, some died of disease progression without receiving any postoperative adjuvant treatment; others died because they did not receive any treatment at all. Therefore, it may be concluded that patients with LCNEO are less likely to receive adjuvant chemotherapy than those with HG-SOC.

Following statistical analysis, we found that patients with LCNEO rarely underwent lymphadenectomy and that more of them (35.2%) no underwent surgery than those with HG-SOC (3.9%). Further, 13.7 and 2.9% of patients in the LCNEO and HG-SOC groups, respectively, were ≥85 years of age. Because patients in the LCNEO group were older, less lymphadenectomy and radical surgery were performed in them; some underwent no surgery at all. It is well-known that satisfactory cytoreduction of ovarian cancer improves patient survival; therefore, we believe that a low rate of cytoreduction affects patient survival and patients with LCNEO are no exception. There were differences in the two groups of patients receiving tumor cytoreduction surgery. We considered the inability to perform satisfactory debulking surgeryor no underwent surgery in the LCNEO group to be due to the advanced age of patients in this group at the time of disease discovery. In addition, we believe that patients with LCNEO were more advanced than those with HG-SOC; therefore, some of them chose palliative care instead of surgical treatment. These factors may also affect the prognosis of patients with LCNEO; therefore, cases need to be accumulated for further analysis and summary in future studies.

Due to its rarity, information regarding LCNEO is limited to case reports and small case series (16–19). The largest report includes one case study and 57 literature reviews (20); 15 of the studies reviewed were on simple LCNEO, while the remaining were on mixed large-cell carcinoma. In this report, the median survival time of patients with LCNEO was 10 months. Although the median survival time of patients in stage I was 48 months, that of patients who had not undergone chemotherapy was only 9.7 months. The disease has a poor prognosis even if diagnosed at an early stage. Most patients received postoperative platinum-based chemotherapy. However, because of the use of platinum-based chemotherapy, the prognosis remains inconclusive. We strongly recommend the establishment of a global LCNEO medical database where international Institutional Clinicopathological Data can be collected and analyzed. Ki et al. (21) reported the case of a patient with LCNEO who survived for only 45 days and that of another patient who had stage Ia disease, underwent six courses of chemotherapy (five after the first course of chemotherapy) with paclitaxel (175 mg/m^2^) and cisplatin (90 mg/m^2^) and given the risk of recurrence within months, also underwent a secondary debulking operation, but unfortunately only survived for 17 months. Lin et al. (17) examined one patient with stage IV disease who survived for only 3 months despite receiving chemotherapy. There are also reports of successful LCNEO treatment. Safini et al. reported the case of a 36-year-old patient with advanced-stage LCNEO who underwent surgery and received adjuvant chemotherapy with etoposide and cisplatin and survived for 4 years (22). In our study, OS and CSS for LCNEO were both related to chemotherapy and tumor size;not receiving chemotherapy and tumors >127 mm were associated with poor prognosis in patients with LCNEO. Our results suggest that chemotherapy should be actively considered for patients with LCNEO. We also observed that while chemotherapy could improve the survival rate of patients with LCNEO, the survival rate of the disease remained low. Our research is based on the SEER database, which provides information on whether patients with LCNEO received chemotherapy but does not specify the type of chemotherapy or number of chemotherapy cycles received. Accordingly, our future research will address this concern.

The diagnosis of LCNEO can be confirmed through immunohistochemistry using one or more standard neuroendocrine-positive markers. The most common markers include synaptophysin, CD56, chromogranin A, neuron-specific enolase, and creatine kinase. Ki et al. (21) reported that increases in the levels of CA125 and NEDD8-activating enzymes are not specific to LCNEO. Similarly, we did not observe a significant difference in CA125 levels between patients with LCNEO and those with HG-SOC in our study.

There is no standardized treatment strategy for LCNEO. Most treatments are performed in line with the recommendations for large-cell lung cancer, including surgical resection and postoperative supplementation with platinum-based chemotherapy regimens (e.g., platinum, paclitaxel, etoposide, and bleomycin) (23). A combination of etoposide and cisplatin/carboplatin or taxol and carboplatin is most commonly used to prolong survival. In the case of pure LCNEO, more consideration should be given to a platinum-etoposide chemotherapy regimen targeting neuroendocrine components (21). However, the 5-year survival rates remain high. Although radiotherapy is more commonly administered in patients with LCNEO than in those with HG-SOC (16), no prospective clinical trials have been conducted to determine its effectiveness in treating LCNEO. We believe that if there are no contraindications to surgery, a combination of surgery and postoperative adjuvant chemotherapy should be considered as the first choice for LCNEO treatment; however, the treatment of LCNEO should be individualized, and the patient’s comprehensive situation should be considered to prolong survival. Individualized treatment plans and principles also require gynecological oncologists to accumulate more cases for further research and exploration.

Previous studies have reported a high probability of metastasis and recurrence even in the early stages of LCNEO (24). LCNEO commonly metastasizes to the pelvic and peritoneal cavities, and metastases to the lungs, brain, and bones are relatively rare (25, 26). Cokmert et al. (27) reported that LCNEO had metastasized to the abdominal skin and limbs at 2 months after surgery. In addition, Agarwal et al. (28) reported the metastasis of LCNEO to the cervix, which is exceedingly rare. A second surgery is often considered to prevent the recurrence and metastasis of LCNEO, and comprehensive postoperative adjuvant treatment is mainly platinum-based. Because cases of pure LCNEO are rare, only a few cases of recurrence have been reported so far. The survival rate of patients with recurrence and metastasis is reportedly very low (1–12 months) (21). Another limitation of the SEER database is that it does not have detailed information on the time and location of recurrence of LCNEO. Therefore, further research cannot be conducted on patients with disease recurrence. Nevertheless, further studies and additional case reports are required to explore factors that can influence LCNEO outcomes and facilitate the development of effective treatment strategies.

This study has several strengths that must be acknowledged. LCNEO is commonly considered a mixed large-cell carcinoma (4–6), and cases of pure LCNEO are rare. An advantage of the SEER database is that it separates pure and mixed large-cell carcinomas, providing us with sufficient data to study simple LCNEO. The SEER database provides information on whether patients with LCNEO received chemotherapy; however, it does not specify the type of chemotherapy or number of chemotherapy cycles received, neither does it contain information on radiotherapy. The database also does not have detailed information on the time and location of LCNEO recurrence; these shortcomings are the limitations of this study. Further research is needed to address these limitations. In addition, to the best of our knowledge, this is the first population-based study comparing LCNEO and HG-SOC, and the data included long-term follow-up records spanning approximately 30 years, increasing the reliability of our analyses. Additionally, propensity-score matching was used to enhance the quality of the analyses by accounting for significant differences in baseline characteristics between the LCNEO and HG-SOC groups. Nevertheless, although our study provides considerable insight and has substantial reliability, the rare occurrence of LCNEO necessitates additional studies with larger sample sizes to draw more convincing conclusions. Therefore, we recommend that a global LCNEO database be used to conduct retrospective and prospective studies designed to identify and develop suitable treatment strategies.

## Conclusion

Given the rarity of LCNEO and lack of systematic population-based research or registration data, there is no consensus regarding its treatment. Clinically, surgical resection is the mainstay, followed by platinum-based chemotherapy. Since the SEER database does not contain information related to disease recurrence or specific radiotherapy and chemotherapy regimens, there are certain limitations to treating patients with LCNEO. In the present study, compared to patients with HG-SOC, those with LCNEO presented more often with advanced-stage disease. Moreover, LCNEO was associated with decreased OS and CSS, compared with HG-SOC. However, based on our analysis of 219 patients, comprehensive treatment may improve the prognosis of patients with LCNEO.

## Data Availability Statement

The datasets presented in this study can be found in online repositories. The names of the repository/repositories and accession number(s) can be found in the article/supplementary material.

## Ethics Statement

The de-identified data in the SEER database are publicly available; thus, their use was exempt from review by the Institutional Review Board of Shengjing Hospital Affiliated with China Medical University. The requirement for informed consent was waived.

## Author Contributions

LP collected clinical data and wrote the paper. ZG helped design and revise the paper. All authors contributed to the article and approved the submitted version.

## Conflict of Interest

The authors declare that the research was conducted in the absence of any commercial or financial relationships that could be construed as a potential conflict of interest.

## Publisher’s Note

All claims expressed in this article are solely those of the authors and do not necessarily represent those of their affiliated organizations, or those of the publisher, the editors and the reviewers. Any product that may be evaluated in this article, or claim that may be made by its manufacturer, is not guaranteed or endorsed by the publisher.

## Abbreviations

AJCC, American Joint Committee on Cancer; CSS, cancer-specific survival; LCNEO, large-cell neuroendocrine carcinoma of the ovary; HG-SOC, high-grade serous ovarian cancer; NSCNEC, non-small-cell neuroendocrine carcinoma; OS, overall survival; SCCO, small-cell carcinoma of the ovary; SEER, Surveillance, Epidemiology, and End Results; WHO, World Health Organization.
